# GABAergic System Dysfunction in Autism Spectrum Disorders

**DOI:** 10.3389/fcell.2021.781327

**Published:** 2022-02-07

**Authors:** Haisheng Zhao, Xijing Mao, Cuilin Zhu, Xiaohan Zou, Fanzhen Peng, Wei Yang, Bingjin Li, Guangquan Li, Tongtong Ge, Ranji Cui

**Affiliations:** Jilin Provincial Key Laboratory on Molecular and Chemical Genetic, Second Hospital of Jilin University, Changchun, China

**Keywords:** autism spectrum disorders, GABA, neurodevelopmental disorders, inhibitory neurotransmission, excitatory/inhibitory balance

## Abstract

Autism spectrum disorder (ASD) refers to a series of neurodevelopmental diseases characterized by two hallmark symptoms, social communication deficits and repetitive behaviors. Gamma-aminobutyric acid (GABA) is one of the most important inhibitory neurotransmitters in the central nervous system (CNS). GABAergic inhibitory neurotransmission is critical for the regulation of brain rhythm and spontaneous neuronal activities during neurodevelopment. Genetic evidence has identified some variations of genes associated with the GABA system, indicating an abnormal excitatory/inhibitory (E/I) neurotransmission ratio implicated in the pathogenesis of ASD. However, the specific molecular mechanism by which GABA and GABAergic synaptic transmission affect ASD remains unclear. Transgenic technology enables translating genetic variations into rodent models to further investigate the structural and functional synaptic dysregulation related to ASD. In this review, we summarized evidence from human neuroimaging, postmortem, and genetic and pharmacological studies, and put emphasis on the GABAergic synaptic dysregulation and consequent E/I imbalance. We attempt to illuminate the pathophysiological role of structural and functional synaptic dysregulation in ASD and provide insights for future investigation.

## Introduction

Autism spectrum disorder (ASD) refers to a series of neurodevelopmental diseases characterized by two hallmark symptoms, social communication deficits and repetitive behaviors (Happe, 1999). Clinical observations suggest that patients with ASD were often comorbid with intellectual disability (ID), mood disorders, epilepsy, and intestinal disorders (Happé and Frith, 1996; Rudolph and Möhler, 2014; Richard et al., 2017). The prevalence of autism has gradually increased over time and with significantly higher frequency in male infants than in female infants (Fatemi et al., 2013). A number of investigations have demonstrated that genetic, epigenetic, environmental perinatal factors and their complicated interaction were implicated in the neurophysiological mechanisms of ASD (Belmonte et al., 2004; Hallmayer et al., 2011; Cattane et al., 2020). However, high clinical heterogeneity challenges both the clinical diagnosis and treatment and further investigations of the cellular and molecular pathogenesis of ASD.

Synaptic neurotransmission, including excitatory and inhibitory neurotransmission, is the neurophysiological basis of various functions of the brain. Aberrant neurotransmission, especially the imbalance of excitation and inhibition in the central nervous system (CNS), might be implicated in the ASD pathogenesis. Based on Genome-Wide Association Study (GWAS) and human genetic evidence, some synaptic genes encoding the synthesis, release, and binding receptor proteins of neurotransmitters have been reported to contribute to the pathogenesis of ASD (Kumar and Christian, 2009). Gamma-aminobutyric acid (GABA) is a pivotal inhibitory neurotransmitter in the CNS. GABAergic inhibitory neurotransmission is critical for the regulation of brain rhythm and spontaneous neuronal activities during brain development. The imbalance between excitation and GABAergic inhibition might be consequently contributed to the dysfunction of the CNS GABAergic system. Increasing evidence has shown that dysfunction of inhibitory neurotransmission is implicated in neurophysiological mechanisms of neuropsychiatry disorders, including schizophrenia, depression, and anxiety (Amin et al., 2006; Möhler, 2012; Duman et al., 2019). Especially, it was widely studied that abnormal neuronal excitatory firing in specific brain regions caused by the absence of GABAergic inhibition underlies the neurophysiological mechanism of epilepsy (Puts and Edden, 2012; Kaila et al., 2014). GABAergic dysfunction might provide a potential reasonable explanation for high comorbidity with epilepsy or increased susceptibility to epilepsy observed in patients with autism (Richard et al., 2017).

In this review, we summarized evidence from genetic, human neuroimaging, postmortem, and genetic and pharmacological studies focusing on GABAergic dysfunction and investigating the consequent excitatory/inhibitory (E/I) imbalance in ASD. We put emphasis on the convergent and neurophysiological alterations of how GABA is involved in ASD from different lines of evidence. We aimed to illuminate the potentiality of GABA and GABA receptor (GABA R) modulators as potential therapeutic targets for ASD.

## Evidence From *In Vivo* Neuroimaging Studies

Human evidence from proton magnetic resonance spectroscopy (1H-MRS), neuroimaging, enzyme-linked immunosorbent assay (ELISA), and postmortem brain studies reported altered levels of GABA metabolites and the ratio of GABA to metabolites, such as glutamate and creatine (Rolf et al., 1993; Dhossche et al., 2002; Thatcher et al., 2009; Al-Otaish et al., 2018). A study showed that the electroencephalogram (EGG) phase reset of 54 patients with ASD showed reduced number and/or strength of thalamo-cortical connections comparing with healthy subjects (Thatcher et al., 2009). Earlier evidence showed increased serotonin levels and decreased levels of amino acids, namely, aspartic acid, glutamine, glutamic acid, and GABA, in patients with autism in comparison with controls (Rolf et al., 1993). However, contradictory results exist that young autism patients exhibited higher plasma GABA levels which decreased with age (Dhossche et al., 2002). Several evidence also reported higher plasma GABA levels and lower glutamate/GABA levels in autistic subjects than in controls (El-Ansary and Al-Ayadhi, 2014; Al-Otaish et al., 2018). Al Otaish et al. (2018) found a higher glutamate/glutamine ratio and lower levels of plasma glutamine in ASD patients. Another study focusing on alterations of anti-neuronal antibody levels including anti-glutamic acid decarboxylase antibodies (GAD), anti-glutamate receptor antibodies, and seven types of anti-ganglioside antibodies levels by ELISA found no significant difference between autistic and control subjects (Bayram et al., 2016).

Recently, amounts of evidence using magnetic resonance spectroscopy (MRS) found that the lower ratio of GABA/glutamate (Harada et al., 2011; Kubas et al., 2012; Drenthen et al., 2016; Ajram et al., 2017), N-acetylaspartate/creatine, GABA/creatine, and glutamate/creatine (Kubas et al., 2012) in the frontal lobes, lower GABA/creatine levels in the anterior cingulate cortex (ACC) of patients with ASD than normal controls (Kubas et al., 2012; Cochran et al., 2015). The binocular rivalry dynamics exhibited in normal controls, which depend on the balance of inhibitory and excitatory cortical dynamics, is absent in participants with autism; these findings indicate reduced GABAergic action in the autistic brain (Robertson et al., 2016). The researchers have drawn a conclusion that children with autism have lower sensorimotor GABA levels *via* a tactile task detecting amplitude discrimination and frequency discrimination under the presence of an adapting stimulus (Puts et al., 2017). ASD patients exhibited lower GABA concentrations in the sensorimotor cortex, and the GABA concentrations were positively correlated with self-reported tactile hypersensitivity in adults with ASD (Sapey-Triomphe et al., 2019). It has been shown that children with autism showed higher GABA concentration in the bilateral visual cortex (VIS) which was found to be related to more efficient search and social impairments (Edmondson et al., 2020). Intriguingly, these results suggest that GABAergic system dysfunction in ASD patients, especially in children, was associated with higher cognitive function, including somatosensory function, search and social behaviors.

A recent investigation found that there was a negative correlation between thalamic GABA and autism-spectrum quotient (AQ) in male participants with autism, while the thalamic GABA levels were positively correlated with AQ in female participants (Fung et al., 2021). These results indicate that GABA levels might correlate with autism symptom severity in a gender-specific way. Considering the high clinical heterogeneity of autism, the altered GABA system might be more pronounced for a specific subtype of clinical ASD. Drenthen et al. found higher Glu/creatine and lower GABA/glutamate concentrations in the high-functioning autism (HFA) participants, which are consistent with previous research results (Drenthen et al., 2016). Evidence showed that children and adolescents with HFA have reduced total N-acetylaspartate (tNAA) and total creatine, increased glutamate +glutamine (Glx)/tNAA, but unchanged Glx and comparable GABA levels between healthy and autism groups (Carvalho Pereira et al., 2018). Intriguingly, they also observed that smaller absolute and relative GABA levels were associated with poor communication skills of patients with autism (Carvalho Pereira et al., 2018). These results indicate a critical relationship of the altered GABA system with clinical autism, but the levels of GABA metabolites and the ratio of GABA to metabolites are not sufficient to be a clinical target for diagnosis due to the poor precision and accuracy.

## Evidence From *In Vitro* Postmortem Studies

Results from postmortem studies have shown that altered GABAergic signaling is involved in the neurophysiological mechanisms of autism. The autistic group had increased GAD 67 mRNA expression in cerebellar interneurons and reduced glutamic acid decarboxylase 65(GAD 65) mRNA in the larger labeled cells in the cerebellar dentate nuclei (Yip et al., 2008, 2009). It suggests that a disturbance of the intrinsic cerebellar circuitry in the autistic brain might affect autism-related cognitive and behaviors by affecting the Purkinje cell (PC) output to other key brain regions. Individuals with autism showed a selective increase in the density of calbindin (CB)+, calretinin (CR)+, and parvalbumin (PV)+ interneurons in the hippocampus than those of normal controls (Lawrence et al., 2010). A recent study using immunohistochemical staining found significantly decreased protein levels of α2 subunit of GABA-A receptor in the axon initial segment of pyramidal cells in supragranular layers of prefrontal cortical areas of autistic participants (Hong et al., 2020). Fatemi et al. reported downregulation of different subunits of GABA-A and GABA-B receptors both in mRNA and protein levels (Fatemi et al., 2009a; Fatemi et al., 2009b; Fatemi et al., 2010; Blatt and Fatemi, 2011; Fatemi and Folsom, 2011). They reported *GABBR1*, *GABBR2*, *GABRA1*, and *GABRB3* were significantly decreased in the cerebellum (Fatemi et al., 2009a; Fatemi et al., 2009b), *GABRA1*, *GABRA2*, *GABRA3*, and *GABRB3* were significantly reduced in the parietal cortex, and *GABRA1* was significantly altered in the superior frontal cortex (Fatemi et al., 2009a).

Evidence from quantitative receptor autoradiographic studies was carried out to determine the density and distribution of excitatory and inhibitory receptors (Blatt et al., 2001; Guptill et al., 2007; Oblak et al., 2009; Lawrence et al., 2010). The binding sites of benzodiazepine, a positive allosteric modulator on GABA-A R, were significantly reduced in the hippocampus and ACC of the autistic brain, while other receptors were not significantly changed (Oblak et al., 2009). Further evidence found that reduced [(3)H]flunitrazepam-labeled benzodiazepines(BDZ) sites in the autistic hippocampus were due to a decrease in the binding site number or number of receptors but not due to the binding affinity (Blatt et al., 2001; Guptill et al., 2007; Lawrence et al., 2010). Besides the hippocampus, the cerebellar region can receive inputs widely from the frontal cortex *via* the granular layer; a significant decrease of BDZ binding sites in the supragranular layers and infragranular layers of the cerebellar region was found (Oblak et al., 2009). These results indicate that a significant dysregulation of the GABAergic system, from the synthesis to receptors, in autistic brains, might provide potential targets for therapeutics and diagnosis of autism and related neuropsychiatric diseases.

## Evidence From Genetic Studies

ASD and other neurodevelopmental disorders which are characterized by repetitive behaviors and social deficits are highly hereditable. Genetic evidence showed that single-gene mutations are rare in families with autism. Each common genetic variant causes a mild genetic effect on ASD risk, while the cumulative effect of common genetic variants contribute up to 15–50% to ASD (Vorstman et al., 2017). Rare genetic variants, including *de novo* and intrinsic variants, contribute 10–30% of the genetic contribution to the occurrence of ASD (Vorstman et al., 2017). Several evidence have observed cytogenetic abnormalities in chromosome 15q11–q13 region in individuals with autism (Buxbaum et al., 2002; Hogart et al., 2007; Guo et al., 2017). 15q11–q13 site contains coding regions of specific subunits of GABA-AR, including *GABRB3*, *GABRA5*, and *GABRG3*, which are strongly implicated in the pathogenesis of autism (Delahanty et al., 2011; Bonnet-Brilhault et al., 2016; Guo et al., 2017). Duplications of gene E3 ubiquitin ligase *UBE3A* and three non-imprinted GABA-AR genes which are located in 15q11.2–q13.1 (Dup15q syndrome) can induce autistic phenotype behavioral deficits (Bonnet-Brilhault et al., 2016). It was found that single-nucleotide polymorphisms (SNP)s of *GABRB3* rs2081648, *GABRA5* rs35586628, and *GABRG3* rs208129 polymorphisms are related to symptom-based behavioral deficits in sociability (Noroozi et al., 2018). *GABRA4* was also found to increase autism risk through interaction with *GABRB1*, indicating a complex gene–gene interaction in GABA receptor subunit genes involved in the ethology of autism (Ma et al., 2005). Thus, overall results suggest that GABA-related gene polymorphisms seem to be sufficient to induce the autistic behavioral phenotype; whether rescue of GABA-related gene deficits by genetic approach can improve behaviors is worth to be further discussed.

## GABAergic Dysfunction in Animal Models of ASD

**FIGURE 1:**
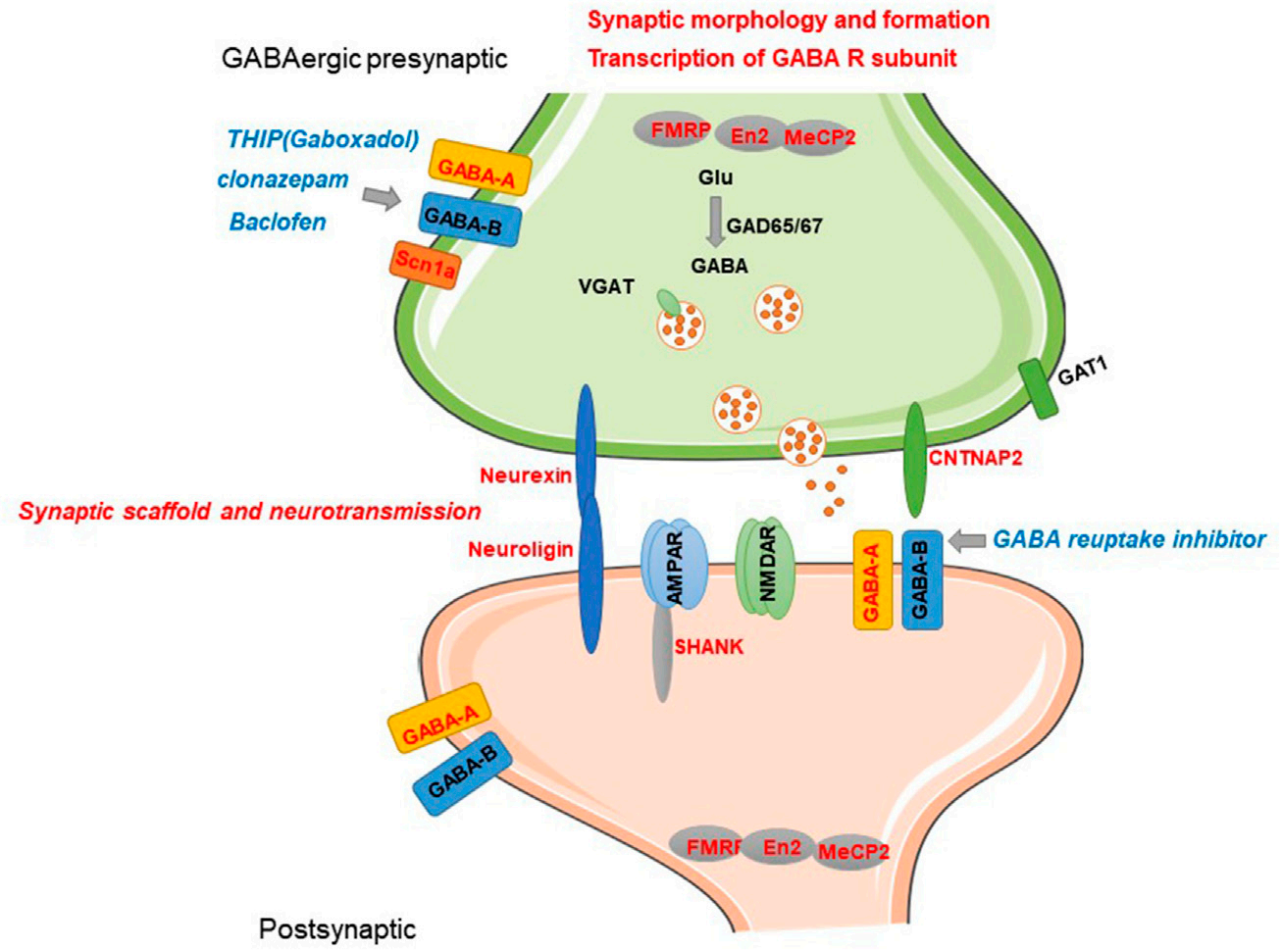
Current known candidate genes of ASD involved in GABAergic synaptic transmission and related mechanisms. Current candidate genes induce dysfunction of GABAergic transmission by affecting transcription of GABA-A subunits, presynaptic GABA release, formation of GABAergic synapse, and synaptic structure–mediated transmission. GABA-related candidate genes and regulatory role are represented by red letters. Pharmacological modulators of GABA-R reported to have alleviation efficacy are represented by blue letters.

Although accumulating *in vivo* and *in vitro* evidence from human studies suggest dysfunction of the GABA system is associated with ASD and genetic evidence further confirmed it, the molecular and cellular mechanisms are still obscure. Recently, transgenic technology enables translating genetic variations into rodent models to further investigate the structural and functional synaptic dysfunction in ASD. Investigating convergent cellular signalings or GABAergic alterations of current genetic and pharmacological animal models contribute to further understanding the mechanisms of ASD. Current candidate genes induce dysfunction of GABAergic transmission by affecting transcription of GABA-A receptor(GABA-AR), presynaptic GABA release, formation of GABAergic synapse, and synaptic structure–mediated transmission (Figure 1).

### GABA Receptor Mutant Animal Models

Direct evidence of GABA dysfunction in autism is from that mouse carrying mutant subunit of GABA receptors exhibited sociability impairments and repetitive behaviors which belong to core symptoms of ASD. Delahanty et al. (2011) first demonstrated that *in vitro* mutant β3 subunit-containing α1β3γ2 or α3β3γ2 GABA-AR showed reduced whole-cell current and functional receptor. *In vivo* evidence showed that a mouse model carrying β3 subunit GABA-AR in which serine residues 408 and 409 (S408/9) mutated to alanines (S408/9A) and S408/9A homozygotes exhibited core ASD symptoms, including increased repetitive behaviors and decreased social interaction, and altered dendrite spine structure and consequently decreased tonic inhibition and increased phasic inhibition (Vien et al., 2015). These results suggest specific GABA-AR defect exert a risk effect linking 15q11–q13 autism duplication region with autism. Impaired GABAergic signaling during brain development is a key event related to the neurodevelopmental disease. In addition, 15q duplication mice (a model for ASD with human chromosome 15q11–q13 paternal duplication) exhibited facilitated activity-induced long-term potentiation (LTP) of glutamate synapses onto layer 5 pyramidal neurons and reduced number of inhibitory synapses, indicating shifted E/I balance is implicated with autism (Saitow et al., 2020).

### Single-Gene Models of ASD

GABAergic dysfunction could also be a downstream consequence of mutations in genes and not directly be involved in GABA transmission because the proper development and function of GABA synapses rely on numerous signaling and scaffolding proteins. Currently known candidate genes of ASD contain transcription factors, scaffolding proteins, receptors, and signaling pathway proteins (Vorstman et al., 2017) (Table 1). Accumulating evidence has shown some of the current candidate genes induce dysfunction of GABAergic transmission by affecting transcription of GABA-AR subunits, development of GABAergic synapse, synaptic structure–mediated transmission. GABA functions as an excitatory neurotransmitter during embryonic development (Ben-Ari, 2002). It was found that prenatal VPA exposure–induced autism model and the fragile X syndrome (Fragile X) ASD model have an abnormal excitatory GABA inhibitory shift during delivery, with elevated intracellular chloride levels and maintenance of higher levels of excitatory GABA, which promotes glutamic acid activity and gamma oscillatory activity (Tyzio et al., 2014). The CA3 hippocampal region of the BTBR T+tf/J mouse, an animal model of idiopathic autism, showed impaired coherent network oscillations at the early postnatal period, which might be attributed to a reduction of the intrinsic excitability of CA3 principal cells and a reduction of the shunting activity in GABAergic interneurons projecting to principal cells (Cellot et al., 2016). These results indicated that the transition of inhibitory to excitatory neurotransmission during early brain development might be pathological mechanisms of imbalance of the E/I ratio and GABA disturbance involved in autism. In contrast, it was found that neurofibromatosis type 1 (NF1) models display increased GABAergic transmission, while decreased GABA levels were observed in patients with ASD (Gonçalves et al., 2017). The inconsistent findings might be due to GABAergic transmission and disproportionately distribution of GABA receptors in a brain region–specific manner. Thus, specific brain regions and developmental time window play a critical role in understanding the mechanisms of GABA involved in autism.

**TABLE 1:** Alterations of GABA system in single-gene models of ASD.

Candidate gene	Animal models	GABA-related mechanisms	Main findings	Reference
*FMR1*	*Fmr1* KO mice	GABA-A subunits	Downregulaton of tonic GABA-A-currents. Downregulation of tonic GABA-A receptor subunits α5 and δ	Curia et al. (2009)
	*Fmr1* KO mice	Amygdalar GABAergic neurotransmission	Reduced amygdalar phasic IPSCs and tonic inhibitory currents	Olmos-Serrano et al. (2010)
	*Fmr1* KO mice	GABA-A subunits	Increased VGAT expression in MNTB	Rotschafer et al. (2015)
			Elevated response thresholds to click and tone stimuli	
	*Fmr1* KO mice	GABA-A subunits	Reduced expression of β1 and δ GABA-AR subunits in mRNA and protein levels in the hippocampus (P22). Reduced expression of α2 GABA-AR subunits in protein levels	Sabanov et al. (2017)
	*Fmr1* KO mice	Hippocampal GABAergic neurotransmission	Reduced hippocampal amplitude of eiPSC and a decrease in the amplitude and frequency of spontaneous IPSCs	Sabanov et al. (2017)
*MeCP2*	*Mecp2* tm1.1Bird]	GABA-A subunits	Reduced VGAT levels. Reduced levels of postsynaptic GABA-AR subunits α2 and α4	Medrihan et al. (2008)
	*Mecp2*-null mice	GABAergic neurotransmission	Unitary responses evoked by minimal stimulation were decreased in the VB but increased in RTN of mutants	Zhang et al. (2010)
	*Mecp2*-null mice	LC GABAergic neurotransmission	Deficient postsynaptic GABAergic inhibition. Decreased frequency and amplitude of the GABA-Aergic mIPSCs in LC.	Jin et al. (2013)
	Mecp2tm1-1Bird	GABA levels	Reduced GABA levels in hippocampus, the midbrain, and the cerebellum	El-Khoury et al. (2014)
	*Mecp2* with A140V mutant	Hippocampal GABAergic neurotransmission	Increased positive resting membrane potential, and increased AP firing frequency induced by injection of depolarizing current in hippocampal CA1 pyramidal neurons	Ma et al. (2014)
	*Mecp2*-null mice	NTS GABAergic neurotransmission; GABA-A subunits	Reduced sIPSC amplitude, sIPSC frequency, and mIPSC amplitude in NTS neurons. Increased expression of GABA-AR δ in the NTS.	Chen et al. (2018)
	*Mecp2+/-* mouse	GABA-AR subunits	Reduced KCC2 and GABA-A1R subunits expression during the pre-symptomatic stage, while the expression was variable in the adult symptomatic mice	Oyarzabal et al. (2020)
*CNTANP*	Cntnap2^−/−^	Number of GABAergic neurons	Reduced number of GABAergic interneurons in all laminae	Peñagarikano et al. (2011)
	*Cntnap4* ^−/−^ mice	GABA-A subunits	Reduced expression of GABA-ARα1 in the hippocampus and thalamus and a reduced trend in the cortex	Heise et al. (2018)
	*Cntnap3*-null mice	Number of GABAergic neurons	Increased GABAergic interneurons in cortical regions and hippocampus *in vivo*	Tong et al. (2019)
	*Cntnap2*-KO mice		Reduced the number of both excitatory and symmetric inhibitory; decreased average amplitude of mEPSCs of pyramidal neurons	Lazaro et al. (2019)
*Neuroligin*	*Nlgn3* R451C knock-in mice	Hippocampal GABAergic neurotransmission	Increased GABAergic drive with enhancement of the frequency of miniature GABA-A–mediated GPSCs on CA3 pyramidal neurons	Pizzarelli and Cherubini, (2013)
	*Nlgn4* KO mice	Hippocampal GABAergic neurotransmission	Altered GABAergic transmission in CA3	Hammer et al. (2015)
	*Nlgn3* R451C knock-in mice	Striatum GABAergic neurotransmission	Impaired LTD at corticostriatal glutamatergic synapses in the dorsal striatum	Martella et al. (2018)
	*Nlgn2* R215H KI *mice*	Hippocampal GABAergic neurotransmission and GABA-A subunits protein levels	Reduced protein levels of subunit GABA-A γ2 and gephyrin in hippocampus. Reduced protein level of PV and VGAT in DG and CA1/CA3 pyramidal cells. Reduced mIPSCs in DG, no changes in mEPSCs	Jiang et al. (2018)
	*Nlgn 2 Arg215 → His215* mutation knock-in *(NL2 R215H KI)* mouse	mPFC GABAergic neurotransmission; GABA-A subunits	Reduced mIPSCs and eIPSCs in mPFC. Reduced levels of PV, subunit GABA-Aγ2, and VGAT in mPFC	Chen et al. (2020)
*SHANK*	*Shank2* knockout mice (e6-7 KO)	GABAergic neurotransmission and GABA-A subunits	Reduced expression of *Gabra2* and GABA receptor-mediated inhibitory neurotransmission	Lim et al. (2017)
	*Shank1* ^*−/−*^ mice	GABAergic neurotransmission	Reduced PV+ neuron-mediated inhibitory synaptic transmission increased E/I balance	Mao et al. (2015)
*EN2*	*En2* ^−/−^ mice	Number of GABAergic neurons	Reduced expression of PV, SOM, and NPY-expressing interneurons in hippocampus and cerebral cortex	Sgadò et al. (2013)
	*En2* ^−/−^ mice	Number of GABAergic neurons	Decreased expression of PV, SST, and *Gabrb3* in the forebrain	Provenzano et al. (2020)

MNTB, medial nucleus of the trapezoid body; eiPSC, equine iPSC; VB, ventrobasal complex; RTN, the reticular thalamic nucleus; SOM, somatostatin; NPY, neuropeptide Y; SST, somatostatin; IPSCs, inhibitory postsynaptic currents; mIPSC, mini-inhibitory postsynaptic currents; mEPSCs, miniature excitatory postsynaptic currents.

#### FMR1

Fragile mental retardation protein (FMRP), encoded by *FMR1*, is an extensively studied RNA-binding protein that is implicated in the pathogenesis of Fragile X, ASD, and other IDs (De Rubeis and Bagni, 2011). Research confirmed *FMR1* and FMRP-targeting gene set associated with ASD and other neuropsychiatry diseases (Jansen et al., 2017). FMRP can affect synaptic phenotype as an mRNA-binding transitional suppressor *via* modulating the synthesis of synaptic proteins which implicated in synaptic transmission (Bagni and Zukin, 2019). Early studies have widely reported altered GABA-AR subunit expression levels in *Fmr1* knockout (KO) mice. Fragile X mice exhibited reduced expression of α1, α3, and α4, β1 and β2, and γ1 and γ2, subunits of GABA-AR both in mRNA and protein levels comparing with wild-type mice. However, GAD65 and GAD67 expression levels were inversely increased in Fragile X mice (D’Hulst et al., 2006; Adusei et al., 2010). It was found that GABA-A β1 and β3, GABAB-R1, two Na^+^–K^+^–2Cl^−^ cotransporter proteins (NKCC1 and NKCC2), gephyrin and ubiquilin, of *Fmr1* KO mice were not significantly different from those of wild-type mice at any of the postnatal time points (Adusei et al., 2010). In addition, *Fmr1* KO mice exhibited abnormal transmission in cognition and sociability-related brain regions. Centonze et al. found that *Fmr1* KO mice have fewer GABAergic synapses whereas higher levels of spontaneous GABA inhibitory transmission in the striatum (Centonze et al., 2008). Phasic GABAergic inhibition was not significantly altered, while the tonic GABA-A currents were downregulated in *Fmr1* KO mice, and this might be related to the lack in expression of GABA-AR subunits α5 and δ in the subiculum (Curia et al., 2009). Furthermore, both significant reduction of the phasic inhibitory postsynaptic currents (IPSCs) and tonic inhibitory currents were observed in the *Fmr1* KO mice. *Fmr1* KO mice showed neuronal hyperexcitability in the amygdala which can be rescued by the administration of GABA agonist gaboxadol (THIP) can rescue the amygdalar neurons *via* promoting the tonic inhibitory tone (Olmos-Serrano et al., 2010). Lack of RGS4, a regulator of G-protein signaling protein, can inhibit the susceptibility of FMR1 mice to audiogenic seizures. Treatment with GABA-B receptor antagonist is prone to induce seizures in *Fmr1/RGS4* double-knockout mice than wild-type mice, whereas agonist of GABA-B baclofen can inhibit seizures in *Fmr1/RGS4* double-knockout mice (Pacey et al., 2009). Similarly, baclofen was found to rescue the increased baseline and auditory-evoked high-frequency gamma (30–80 Hz) power in *Fmr1* KO mice and improved the impaired working memory (Sinclair et al., 2017). These findings indicate impaired GABAergic transmission, especially in cognition and sociability-related brain regions, appears to be implicated in the contribution of gene *FMR1* in neurodevelopmental and neuropsychiatry disorders.

#### MeCP2

MeCP2 is a structural chromosomal protein encoded by gene *MeCP2* which is implicated in Rett syndrome (RTT) pathology (Amir et al., 1999) and subsequently was found to cause non-specific ASD and neurodevelopmental diseases (Tillotson and Bird, 2019). The loss of MeCP2 from a subset of forebrain GABAergic neurons also recapitulates many features of RTT (Chao et al., 2010). Earlier evidence showed that *MeCP2*-deficient GABAergic neurons of forebrain showed reduced inhibitory quantal size, which is consistent with a presynaptic reduction in GAD1 and GAD2 expression levels, and GABA immunoreactivity (Chao et al., 2010). It was found that MeCP2 disruption causes the defect of GABA release from presynapse and impaired GABAergic postsynaptic inhibition in locus coeruleus (LC) and NTS (Jin et al., 2013). *MeCP2*-null mice exhibited increased amplitude of spontaneous miniature and evoked excitatory postsynaptic current (EPSC)s without affecting the intrinsic excitability in the nucleus tractus solitarius (NTS), which is associated with decreased brain-derived neurotrophic factor (BDNF) availability in the primary afferent pathway, and these changes can be normalized by exogenous BDNF application (Kline et al., 2010).

Some evidence has demonstrated that the loss of MeCP2 induces reduced expression levels of vesicular GABA transporter (VGAT), GAD1, and GAD2, and altered expression of GABA-AR subunits (Medrihan et al., 2008; Chao et al., 2010; Chen et al., 2018; Oyarzabal et al., 2020). In particular, it was found that GABA-A subunit expression was developmentally decreased especially in the no-symptom stage in *MeCP2*+/− mouse brain (Oyarzabal et al., 2020). MeCP2 loss decreased the level of spontaneous glutamate receptor-mediated synaptic currents in hippocampal CA3 neurons and induced hyperexcitability in hippocampal neurons (Zhang et al., 2008; Calfa et al., 2011; Ma et al., 2014). Direct evidence was found from studies demonstrating that neurons lacking MeCP2 exhibited the defect of GABAergic transmission whereas not in the neighboring neurons with MeCP2 and forebrain-restricted loss of Mecp2 reduce the number of GABAergic synapses in the cortex (Zhang et al., 2014). These results indicate that MeCP2 loss induces defect in presynaptic GABA release, reduced number of GABAergic synapses, and impaired GABAergic inhibition.

Pharmacological stimulation of the GABAergic system exhibited restoration of abnormal neurotransmission and behavioral phenotype. GABA-A-R agonist THIP (gaboxadol) was reported to alleviate symptoms of RTT, including the motor dysfunction and social abnormalities (Zhong et al., 2017). GABA reuptake inhibitor can improve the life span of *Mecp2*-deficient mice (El-Khoury et al., 2014). Application of benzodiazepine-like modulator midazolam can transiently abolish the breathing defects of *Mecp2*-deficient mice (Voituron and Hilaire, 2011). Notably, mirtazapine, an antidepressant known to promote GABA release, was found to inhibit the hopping behaviors of *Mecp2*-null mice (Bittolo et al., 2016). These results confirmed that GABAergic neurotransmission defect appears to be involved in neurophysiological mechanisms of MeCP2 deficiency–induced abnormal behaviors.

Notably, sex difference of RTT has higher prevalence in girls than in boys and is significantly distinguished with ASD (Beattie, 2004). It was shown that RTT is the second most prevalent cause of intellectual disability in girls (Matagne et al., 2017). The maternal experience transiently enhances GAD67 in the auditory cortex and enhances PV-expressing neurons only in *MeCP2* het mice; knockout of *MeCP2* specifically in PV neurons is sufficient to impair learning behavior of pups, indicating experience-dependent plasticity might be impaired during development in adult *MeCP2* het mice (Krishnan et al., 2017). Only restoration of the MeCP2 expression in GABAergic neurons of male *MeCP2* null mice can increase inhibitory neurotransmission and subsequently improve ataxia and social impairments but not anxiety; these physiological and behavioral changes were not observed in female mice (Ure et al., 2016). These results suggest that MeCP2 is critical for the regulation of the GABAergic system, especially during neurodevelopment.

#### Nlgn2, 3, and 4

Neurexins (NRXNs) and neuroligins (NLGNs) are critical structural molecules involved in synapse formation and differentiation as cell adhesion molecules. NRXNs localized in presynapse can bind to postsynapitc NLGNs, bridge the synaptic cleft, and contribute to synapse stabilization (Blunk et al., 2014). *In vivo* and *in vitro* evidence have demonstrated that neuroligin-2 plays an important role in the development and maintenance of GABAergic synapses (Chen et al., 2011; Varley et al., 2011; Fekete et al., 2015; Uchigashima et al., 2016; Panzanelli et al., 2017). NLGN2 can precede KCC2 expression and regulate GABA functional switch in early postnatal development (Panzanelli et al., 2017). Notably, knockdown of *Nlgn2* can reverse the inhibitory GABA to excitatory action in mature neurons, indicating NLGN2 involvement in maintenance of GABA function as an inhibitory neurotransmitter in mature neurons (Sun et al., 2013). NLGN2 can induce fast decay of mini inhibitory postsynaptic currents (mIPSC) by regulation of differential expression of postsynaptic GABA-AR subtypes (Fu and Vicini, 2009). Overexpression of NLGN1B and NLGN2A in newborn neurons of the hippocampus can increase the spine density and impaired spatial memory (Krzisch et al., 2017). These data suggest that neuroligins might affect the formation of neural connectivity *via* affecting the early postnatal transition of excitatory GABA.

Several evidence have shown that abnormal NLGN2 expression is associated with neuropsychiatric behaviors. Reduced NLGN2 expression in the prefrontal cortex (PFC) is involved in attention deficits induced by peripubertal stress which can be reversed by virus-mediated NLGN2 rescue (Tzanoulinou et al., 2016). Animal models using the genetic engineering method showed that *Nlgn 2* R215H knock-in (KI) mouse exhibited schizophrenia-like behaviors and higher anxiety behaviors with significant reduction of mIPSCs in the dentate gyrus (DG), reduced subunit GABA-A γ2 expression levels, and reduced presynaptic PV and VGAT levels, while there were no changes of miniature excitatory postsynaptic currents (mEPSCs), vesicular glutamate transporter (VGLUT), and postsynaptic density protein 95 (PSD95) expression levels (Jiang et al., 2018; Van Zandt et al., 2019). NLGN2 of ACC is involved in KCC2-mediated abnormal GABAergic transmission induced aggressive behaviors (Jager et al., 2020).

Social deficits, enhanced spatial learning, increased locomotor activity, and increased aggressive behaviors were observed in *Nlgn3* R451C mutant mice (Jaramillo et al., 2014; Burrows et al., 2015). It was found that elevated aggressive behaviors in *Nlgn3* R451C mutant mice could be normalized by risperidone, a novel antipsychotic agent that antagonizes serotonin (5-hydroxytryptamine) 5-HT2 and dopamine D2 receptor (Burrows et al., 2015). Earlier findings demonstrated *Nlgn3* R451C mutant mice exhibited enhanced GABAergic transmission with increased frequency of IPSCs while having no significant effect on excitatory transmission (Tabuchi et al., 2007). Both *Nlgn3* R451C knock-in and Nlgn3 KO mutant impaired tonic endocannabinoid signaling (C et al., 20132020). *Nlgn3* R451C knock-in mice appeared to show an increased GABAergic drive with enhancement of the frequency of miniature GABA-A–mediated giant depolarizing potentials (GPSCs) on CA3 pyramidal neurons (Pizzarelli and Cherubini, 2013). In addition, mice carrying R451C-*Nlgn3* mutation showed impaired long-term depression (LTD) at corticostriatal glutamatergic synapses which could be rescued by exogenous activation of cannabinoid CB1 receptors or enhancement of the endocannabinoid tone (Martella et al., 2018). Mice and rats carrying *Nlgn3* mutations showed reduced striatal glutamate indicating glutamate/GABA abnormalities in the corticostriatal circuitry (Horder et al., 2018).

Hippocampal *Nlgn*4 likely localized at excitatory synapses originating from PV-positive interneurons (Marro et al., 2019). ASD-related *Nlgn*4 mutations can alter the compositions of hippocampal perisomatic inhibitory synapses and impaired the synchronized oscillatory activity which might be implicated in the cognitive dysfunction of ASD (Hammer et al., 2015). Glycine is another widely acknowledged inhibitory neurotransmitter in the CNS. It was found that *Nlgn4* KO mice exhibited impaired glycinergic transmission in the retina with fewer glycine receptors mediating fast glycinergic transmission and slower glycinergic mIPSCs (Hoon et al., 2011). A recent study confirmed that NLGN4 significantly impaired glycinergic transmission and reduced the numbers of glycinergic synapses while having no impact on glutamatergic synapses. Intriguingly, they found overexpression of NLGN4 causes impairments of excitatory transmission but not that of inhibitory transmission (Zhang et al., 2018). These findings confirmed the contribution of members of NLGN family to neurodevelopmental diseases *via* various molecular and cellular mechanisms.

#### CNTNAP2, 3, and 4

*CNTNAP2* encodes contactin-associated protein-like 2 (Caspr2), a protein of the neurexin superfamily, and interacts with NLGN proteins, scaffolding proteins PSD95 and gephyrin (Bonnet-Brilhault et al., 2016). Human and animal studies have reported that mutations in *CNTNAP2* cause a syndrome form of ASD with significant behavioral defects in social interaction and stereotypes (Peñagarikano et al., 2011). It was found that *Cntnap2* ^−/−^ mice showed ASD core symptoms, accompanied with significant reduced GABAergic neurons and reduced cortical neuronal synchrony; these changes can be rescued by risperidone treatment (Peñagarikano et al., 2011). *Cntnap2* KO mice displayed remarkable reduced excitatory and inhibitory synaptic inputs onto pyramidal neurons of mPFC with normal dendritic arborization, and decreased average amplitude of mEPSCs in pyramidal neurons, while no significant changes in mIPSCs (Lazaro et al., 2019). However, contradictory results from a genome analysis found that rare heterozygous mutations in any of the *CNTN* or *CNTNAP* genes, including *CNTNAP2*, were not significantly involved in the plausible contribution to risk of autism (Murdoch et al., 2015).

Other members of CNTNAP family are reported to be related to autism (Tong et al., 2019). It was found that knockdown of *CNTNAP3* expression led to the decreased dendritic length and branch. Intriguingly, the formation of excitatory synapse in CNTNAP3-knockdown mice was decreased, while that of the inhibitory synapse was increased (Tong et al., 2019). ASD-related mutations in *CNTNAP3* (R1219X, P614A, and R786C) may be a loss-of-function mutation (Tong et al., 2019). Earlier evidence showed that *CNTNAP4* might be relevant in autism (Cottrell et al., 2011). CNTNAP family contained five members from CNTNAP1 to CNTNAP5, while how CNTNAP5 exert function was obscure. These results indicate that CNTNAP has extensive effect on synapse formation and function; the specific molecular mechanisms of CNTNAP involved in synapse function and ASD need to be further investigated.

#### SCN1A

Haploinsufficiency of the *SCN1A* gene encoding brain voltage-gated sodium channel NaV1.1. is the genetic etiology of Dravet syndrome, a developmental disorder characterized by recurrent intractable seizures, cognitive deficit, and autistic-like behaviors (Han et al., 2012). Han S et al. reported that haploinsufficiency of the *SCN1A* gene impaired GABAergic neurotransmission by conditional deleting the NaV1.1 channels in forebrain interneurons which can be fully rescued by low-dose treatment with clonazepam, a positive allosteric modulator of GABA-AR (Han et al., 2012). The expression of the *SCN1A* gene was found significantly decreased in PFC of offspring of maternal immune activation (MIA) models, which also indicates impaired GABAergic transmission involved in pharmacological models of autism (Yang J. Q. et al., 2021).

#### SHANK1, 2, and 3

The Shank family of scaffold proteins, mainly containing members *SHANK1 to SHANK3*, can bind a variety of membrane and cytoplasmic proteins to form a postsynaptic scaffolding protein complex which is related to the formation and function of excitatory synapses (Sheng and Kim, 2000). Though *de novo* deletions, insertion, splicing mutations, and point mutations of *SHNAK* have been identified in ASD patients, *in vivo* mechanisms in which *SHANK* relate to ASD remain obscure. Animal evidence showed absence of shank altered to the expression of receptor subunits of GABA and GABAergic inhibitory synaptic transmission. SHANK1 protein is reported to be highly expressed in PV-positive fast-spiking inhibitory interneurons in the hippocampus (Mao et al., 2015). *Shank1* mutant mice have impaired inhibitory synaptic outputs to hippocampal CA1 pyramidal neurons and consequently impaired excitatory and inhibitory balance (Mao et al., 2015). *Shank1−/−* and *Shank3B−/−* mice showed reduced PV expression in mPFC and somatosensory cortex and striatum (Filice et al., 2016). *Shank2* knockout mice (e6-7 KO) showed a reduction in the expression levels of *Gabra2* and GABA R-mediated inhibitory signaling, which can be reversed by treatment with an allosteric modulator for the GABA-AR (Lim et al., 2017). *Shank2−/−* and *Shank3*αβ*−/−* mice exhibited reduced levels of several cell surface glutamate receptors, such as GluN1 and GluA2, in the striatum and cortex (Heise et al., 2018). Considering that SHANK family proteins are mainly localized at the postsynaptic densities of excitatory synapses, there is still lack of evidence showing a direct relationship between SHANK and GABAergic transmission.

#### EN2

Several evidence have shown homeobox transcription factor Engrailed2 (En2) appears to be a susceptibility locus for ASD (Benayed et al., 2005; Brune et al., 2008; Wang et al., 2008; Benayed et al., 2009). Mouse carrying mutants in *En2* exhibited similar morphological abnormalities in the cerebellar with autistic individuals (Gharani et al., 2004). Protein EN2 is mainly present in postnatal GABAergic neurons of the mouse hippocampus and cerebral cortex, and it was found that persistant high expression of EN2 during the prenatal and early postnatal period contributes to autism by affecting the maturation of Purkinje cell (PCs) in the cerebellum (James et al., 2013, 2014). EN2 expression levels alter the number of GABAergic interneurons (Sgadò et al., 2013; Provenzano et al., 2020). Evidence from an *in vitro* study found that En2 increases the complexity of the dendritic tree of GABAergic neurons and reduced the number of mature synapses as well as the area of postsynaptic densities (Soltani et al., 2017).

### Pharmacological Models

Besides the single-gene models, pharmacological models were widely used to discuss the pathogenesis of ASD and identification of novel drug targets (Table 2). Valproic acid (VPA) as a broad-spectrum antiepileptic drug has a significant facilitative effect on the GABAergic neurotransmission system (Miyazaki et al., 1988; Lattanzi et al., 2019). Intraperitoneal administration of VPA can significantly increase GABA levels and upregulate the seizure thresholds (Nau and Löscher, 1982; Löscher and Vetter, 1984). Amounts of evidence have shown that prenatal exposure with 400–600 mg/kg VPA was found to induce significant autistic behaviors in offspring which was widely used as pharmacological rodent models for the ASD study (Nicolini and Fahnestock, 2018). Earlier evidence has shown that VPA prenatal exposure-induced ASD models have a significant reduction of PV+ inhibitory interneurons numbers in the parietal and occipital cortex (Gogolla et al., 2009), reduced GABA-A and GABA-B receptor expression, and reduced GABA levels (Kazlauskas et al., 2016), reduced GAD65 and GAD67 levels in the cerebellar, amygdala (Olexová et al., 2016; Chau et al., 2017), and cortex (Cusmano and Mong, 2014; Chau et al., 2017; Hou et al., 2018), and reduced GAT1 expression in the amygdala (Olexová et al., 2016). In contrast, contradictory results found that cortical GAD67 expression levels showed an increasing trend from adolescence to adulthood in VPA models (Hou et al., 2018). High-performance liquid chromatography (HPLC) results showed that the levels of glutamate, Gln, and GABA were significantly increased in the hippocampus of male VPA and thalidomide (THAL)-induced autism rats. Nuclear magnetic resonance (NMR) spectroscopy results showed increased levels of Gln and GABA in the VPA models (Zieminska et al., 2018). A recent study reported widely decreased expression of genes encoding different subunits of GABA-AR, including *GABRA1*, *GABRA2*, *GABRA3*, *GABRB3*, *GABBR1*, and *GABBR2* (Yang Y. et al., 2021), which is consistent with an earlier study reporting reduced *GABRA1*, *GABRA5*, and *GABRB2* levels in the cortex of VPA-induced autism models (Chau et al., 2017). Yang et al. found that the frequency and amplitude of spontaneous excitatory postsynaptic currents (sEPSCs) were comparable between control and VPA mice models, while the frequency of spontaneous inhibitory postsynaptic currents (sIPSCs) was decreased in VPA models (Yang J. Q. et al., 2021). It was reported that the normal expression of GABAρ3 in the cerebellum was increased linearly throughout the normal development process, while the VPA models exhibited reduced GABAρ3 levels in PCs and ependymal glial cells (EGCs) from lobule X of the cerebellum (Varman et al., 2018). Administration of both GABA-A and GABA-B receptor agonists can improve the social deficits (Kazlauskas et al., 2016; Yang Y. et al., 2021). Individual GABA-AR agonist, chlorpromazine or GABA-BR baclofen administration can alleviate autistic-like behaviors in the VPA model. Combined chlorpromazine with baclofen can alleviate sociability deficits, anxiety and repetitive behaviors (Yang Y. et al., 2021). This evidence suggests impaired GABAergic inhibitory transmission in prenatal VPA exposure- induced autism models.

**TABLE 2:** Alterations of the GABA system in pharmacological models of ASD.

Pharmacological models	Method	GABA-related mechanisms	Main findings	Reference
VPA models	C57BL/6 mice (E 10.5 500 mg/kg i.p. injection	Number of GABAergic neurons	PV-positive GABAergic neurons were reduced in the neocortex	Gogolla et al. (2009)
	BALB/c mice (E 12.5 600 mg/kg i.p. injection)	GABA-related proteins	Excitatory synaptic proteins NR2A, NR2B, NR2C, and inhibitory-related proteins GAD65, GAD67, GABRA1, GABRA5, and GABRB2 were downregulated	Chau et al. (2017)
	Rats (E12.5 600 mg/kg i.p. injection	GABA-related proteins	GAD65, GAD67, and GAT1 were evaluated in the amygdala; GAD65 and GAD67 mRNA were decreased in the cerebellum	Olexová et al. (2016)
	Sprague–Dawley rats (E12.5 600 mg/kg i.p. injection	GABA-related proteins	GAD67 was decreased in HP and cerebellum, and temporal cortex, and was increased in PFC.	Hou et al. (2018)
	Sprague–Dawley rats (E12.5 400 mg/kg i.p. injection	GABA-related proteins	GAD65 and GAD67 were decreased in cortical tissue. Increased high-frequency activity during wake and REM sleep	Cusmano and Mong, (2014)
	Wistar rats (VPA, 800 mg/kg b.w)	GABA neurotransmitter levels	*Ex vivo* HPLC studies Glutamate, Gln, and GABA significantly increased in male rat’s hippocampus. NMR studies showed increased levels of Gln and GABA in the VPA group	Zieminska et al. (2018)
	CD-1 strain or GFAP-eGFP transgenic mice (E12.5, 500 mg/kg i.p. injection	GABA receptor subunit expression pattern	GABAρ3 in PCs and EGCs from lobule X were reduced. GABAρ3 expression increases linearly throughout normal development of the cerebellum. GABAρ3 expression is disrupted in the VPA models	Varman et al. (2018)
MIA models	C57BL/6 mice (E12.5 20 mg/kg of poly (I:C), i.p. injection	GABAergic neuron phenotype shift	Scn1a mRNA levels were not significantly changed; protein levels of Na_V_1.1 were decreased in the mPFC	Yang et al. (2021a)
	C57BL/6 mice (E17 5 mg/kg intravenous (iv)	GABAergic neuron phenotype shift; GABA-related protein expression	Increase in the transcription of NKCC1 and decrease in KCC2. Reduced mRNA expression levels of GAD65, GAD67, and VGAT	Richetto et al. (2014)

REM, rapid eye movement; EGCs, ependymal glial cells; PCs, Purkinje cells.

Another widely studied pharmacological model of ASD is the MIA model, which refers to immune activation by immunogen administration during pregnancy, such as lipopolysaccharide (LPS) or polyinosinic–polycytidylic acid (poly (I:C)) (Meyer, 2014; Haddad et al., 2020). The mRNA and protein levels of GAD65, GAD67, and GABA-A α2, 3, 4, 5 were reduced in the cortex of MIA models, and there was no significant difference in expression levels of GABA and glutamate-related subunits in the cortex or cerebellum (Zhang et al., 2021). Treatment with low-dose benzodiazepines clonazepam can completely reverse behavioral abnormalities of offspring of MIA models by enhancing GABAergic neurotransmission (Yang J. Q. et al., 2021). In addition, the mRNA expression levels of NKCC1 and KCC2 were increased in the peripubertal offspring of MIA models (Richetto et al., 2014). The presence of imbalanced NKCC1 and KCC2 expressions in the adult brain is typically taken as an index of an immature GABAergic phenotype (Ben-Ari et al., 2012). Thus, these results suggest prenatal maternal immune activation might have effect on long- lasting GABAergic changes relevant to autism and other neuropsychiatric disorders with prenatal infectious etiologies.

## Potential Therapeutic Efficacy of GABA R Modulators in ASD

GABA-A receptors are a series of ion channel–type receptors that are sites of action for benzodiazepines, barbiturates, and anesthetics. GABA-B receptors are metabotropic receptors (Jembrek and Vlainic, 2015). Presynaptic GABA-B receptors act by inhibiting voltage-gated calcium channels (Krystal et al., 2002). As mentioned above, accumulating evidence has shown some of the current candidate genes mutant induce dysfunction of GABAergic transmission by affecting transcription of GABA-A subunits, development of GABAergic synapse, and synaptic structure–mediated transmission. Treatment with GABA agonists can alleviate autism-like behaviors by disinhibition of presynaptic GABAergic neurons. In addition, diuretic NKCC1 chloride importer antagonist bumetanide can reinforce GABAergic inhibition by decreasing Cl-; animal evidence showed that treatment with bumetanide reduces the severity of autism symptoms (Hadjikhani et al., 2018; James et al., 2019). The most widely studied GABA modulator is baclofen, a GABAB receptor agonist, which was reported to reverse social approach deficits and repetitive behaviors of BTBR T+ Itpr3tf/J (BTBR) mice, and also reduced the stereotyped jumping in C58/J (C58), another ASD model (Silverman et al., 2015). In addition, the hyperactivity, anxiety, aggression, and repetitive behaviors of the *FMR1* KO mouse model were found to be completely rescued by a GABA-AR agonist, gaboxadol, which is also called OV101 or THIP and prefer to activate δ subunit-containing extrasynaptic GABA-AR (Cogram et al., 2019).

Up to now, there are several positive results from clinical trials on the role of GABA modulators on patients with autism. Brondino et al. summarized human clinical trials of pharmacological efficacy of GABA modulator on autism (Brondino et al., 2016). A randomized, controlled, phase 2 trial results showed arbaclofen failed to improve the abnormal behavioral phenotype of children and adults with fragile X syndrome (Berry-Kravis et al., 2012). A randomized, placebo-controlled, phase 2 study of 150 participants with ASD showed there was no difference in the primary outcome measure between the arbaclofen group and control group, while there was improvement on the clinician-rated clinical global impression of severity in a specified secondary analysis (Veenstra-VanderWeele et al., 2017). Baclofen as an adjuvant therapy can enhance the therapeutical effect of antipsychotic drugs, risperidone, in children with ASD, including improvement of irritability, lethargy, stereotypic behavior, hyperactivity, and inappropriate speech (Mahdavinasab et al., 2019). A study with a small sample found that treatment with bumetanide decreased the autistic behaviors in autistic infants (Lemonnier and Ben-Ari, 2010). In addition, an open-label trial pilot study showed 10 months of bumetanide treatment can improve emotion recognition and stimulate the activation of social and emotional perception-related brain regions during the perception of emotional faces in adolescents and young adults with autism (Hadjikhani et al., 2015). A recent investigation showed bumetanide significantly reduces the severity of ASD core symptoms which relate to a decreased GABA/glutamate ratio in both the insular cortex (IC) and visual cortex (VC) (Zhang et al., 2020). In conclusion, current evidence in clinical trials is limited by small sample sizes and short study follow-ups, complicated clinical heterogeneity, and inconsistent inclusion criteria. Thus, to date, there is insufficient evidence to suggest the use of GABA modulators in autistic patients. Medication safety is also an issue to consider in addition to efficacy.

## Conclusion and Future Perspectives

Numerous ASD candidate genes regulate extensive functions of the CNS; thus, it is hard to converge specific molecular mechanisms of ASD according to current genetic and pathological evidence. However, a lot of ASD-related genetic variations from human genetic studies identified the involvement of synaptic genes which consolidate the hypothesis that synaptic structure or/and function dysfunction are involved in ASD. In addition, technology advances propel modeling rodent models of ASD and other neuropsychiatric disorders using manipulation of candidate genes or environmental factors. We summarized primary GABAergic inhibitory dysfunction of animal models of non-syndromic gene mutations (CNPANP, neuroligin, SHANK, and EN2) and syndromic gene mutations (FMR1, Mecp2, and SCN1A). Current evidence indicates GABA is related to ASD from two aspects. First, GABA receptor mutants in specific neurons sufficiently recapitulate core ASD symptoms. Duplications of gene E3 ubiquitin ligase UBE3A and three non-imprinted GABA-AR genes located in 15q11.2–q13.1 (Dup15q syndrome) induce autistic phenotype behavioral deficits (Bonnet-Brilhault et al., 2016). Besides, well-acknowledged ASD candidate genes were specifically expressed in GABAergic neurons and implicated in GABA function. Scn1a encodes sodium channel subunits that are upregulated in Pv+ fast-spiking interneurons. Pharmacological approaches promoting GABA transmission suppress behavioral symptoms of Dravet’s syndrome (Han et al., 2012). The structural chromosomal protein MeCP2, homeobox transcription factor En2, and RNA-binding protein FMRP target metabolism, synthesis and modification of synaptic proteins related with GABA synaptic function. Neuroligins might affect the formation of neural connectivity via affecting the early postnatal transition of excitatory GABA.

Pharmacological intervention with agonist targeting specific GABA receptors was found to partially alleviate ASD-related behavioral abnormalities in animal models and clinical trials. However, the therapeutic efficacy of clinical modulators of GABA receptors needs to be supported by clinical trials with larger sample sizes. These results collectively suggest that GABAergic synaptic transmission dysfunction, especially in specific neurons or circuitry during the developmental stage, might underlie the pathophysiology of ASD. However, increased GABAergic synaptic transmission is also reported in some animal studies, indicating impaired GABAergic transmission might be a critical etiology of imbalance of the E/I ratio involved in the development of ASD. Substantial questions as to the specific etiology of ASD remain unclear and require future investigation. First, researchers need to take the reliability of current behavioral assays of mice into consideration when mimicking the clinical ASD phenotype. Additionally, a complicated reciprocal relation exists between the excitation and inhibition, especially in early brain development. In consideration that GABA can shift from “excitatory” to “inhibitory” and play a critical role in subtle regulation of neuronal network during critical developmental stages, it cannot be easily ruled out that altered excitation might be secondary to reduced inhibition and GABA dysfunction. Thus, further investigations are needed to shed light on the pathogenesis of ASD and provide a comprehensive understanding of synaptopathology in ASD.
